# Exploring the link between environmental chemical exposures and epigenetic modifications in diabetes mellitus: A review

**DOI:** 10.17305/bb.2025.11801

**Published:** 2025-05-01

**Authors:** Tasnim R Matarid, Menatallah Rayan, Ola J Hussein, Hanan H Abunada, Zaid H Maayah, Hesham M Korashy

**Affiliations:** 1 Department of Pharmaceutical Sciences, College of Pharmacy, QU Health, Qatar University, Doha, Qatar; 2 Biomedical Research Center, QU Health, Qatar University, Doha, Qatar

**Keywords:** Diabetes mellitus, insulin resistance, glucose metabolism, environmental pollutants, aryl hydrocarbon receptor, epigenetic modifications

## Abstract

Diabetes mellitus (DM) is a globally prevalent metabolic disorder characterized by impaired glucose homeostasis and insulin secretion. Beyond traditional risk factors like lifestyle and genetics, environmental pollutants, including particulate matter, heavy metals, and persistent organic pollutants, have become significant contributors to DM. One of the key mechanistic pathways through which these pollutants exert their effects is the activation of the aryl hydrocarbon receptor (AhR), a ligand-activated transcription factor that regulates the expression of cytochrome P450 family 1 (CYP1) enzymes. This cascade contributes to increased oxidative stress and systemic inflammation, hallmarks of metabolic impairment. Importantly, these environmental pollutants are also linked to epigenetic modifications, including aberrant DNA methylation, histone modifications, and microRNA dysregulation, which further disrupt insulin sensitivity and β-cell function. This review explores the possible mechanistic crosstalk between AhR/CYP1 pathway activation and epigenetic alterations in the context of diabetes development. By integrating findings from epidemiology, *in vivo*, and *in vitro* studies, we provide a summary of how environmental exposures may influence diabetes risk through epigenetic mechanisms. Understanding these interactions not only advances our knowledge of DM etiology but also highlights novel molecular targets for preventive and therapeutic strategies.

## Introduction

Diabetes mellitus (DM) is one of the most burdensome and widespread metabolic disorders. It has reached epidemic proportions globally, with a significant increase in prevalence leading to considerable impacts on individuals’ quality of life. If untreated or poorly managed, DM contributes to both short- and long-term complications [1]. According to the latest World Health Organization statistics, about half a billion people worldwide suffer from DM. Approximately 1.5 million deaths each year are attributed to DM, with the majority occurring in low- and middle-income countries. The prevalence of DM has steadily increased over recent decades and is projected to exceed 550 million by 2030 [1]. DM occurs in two forms: type 1 DM (T1DM) and type 2 DM (T2DM) [2].

T1DM is a chronic autoimmune disease in which activated T lymphocytes mediate an autoimmune response, attacking and destroying pancreatic beta cells (β-cells), mistakenly recognizing them as autoantigens. T1DM is characterized by very little to no insulin secretion by the pancreas [2] and accounts for around 10% of DM cases globally, with the highest incidence observed among younger individuals aged 10–14 years [3]. Epidemiological studies have highlighted that family history, age, gender, dietary habits, and other factors such as viral infections contribute to T1DM [2, 4]. Uncontrolled T1DM can result in diabetic ketoacidosis, a life-threatening condition characterized by fruity-smelling breath, visual disturbances, labored breathing, and unconsciousness [5]. On the other hand, T2DM is the most common form of DM and accounts for roughly 90% of cases [6]. T2DM is a chronic metabolic disorder in which the pancreas secretes an insufficient amount of insulin in response to systemic insulin resistance [2]. It is characterized by elevated blood glucose levels due to reduced insulin sensitivity in the liver and peripheral tissues, such as skeletal muscles and adipose tissues. Moreover, β-cell function declines over time due to prolonged exposure to associated risk factors and elevated glucose levels, which impair insulin secretion. Both genetic and non-genetic factors, such as a sedentary lifestyle, obesity, and aging, are associated with an increased risk of T2DM [7, 8]. Poorly managed T2DM can damage both microvascular and macrovascular systems, leading to long-term complications, such as retinopathy, neuropathy, and nephropathy [6].

Despite extensive research in the field of DM and the identification and characterization of traditional risk factors, these conventional factors are insufficient to explain the sharp global increase in cases, suggesting that additional risk factors may contribute to this rise. Among these, exposure to environmental toxins, such as pollutants and chemicals has been shown to play a significant role in the development and progression of various diseases, including cancer [9, 10], autism [11], and cardiovascular disorders [12]. Recently, Sayed et al. [13] reviewed the physiological and pathological roles of environmental toxins in glucose homeostasis and insulin resistance. Although the review highlighted some of the molecular mechanisms involved—such as gluconeogenesis, hypoxia-inducible factor, oxidative stress, and inflammation [13]—the impact of exposure to environmental chemicals and pollutants on epigenetic modifications in DM remains largely unexplored. Therefore, the current review focuses on understanding and further exploring potential molecular mechanisms and the emerging impact of risk factors, particularly the influence of environmental pollutants and epigenetic modifications on the development of diabetes and insulin resistance.

## Environmental pollution and DM

The impact of environmental pollutants on human health has become an increasing concern, particularly since the Industrial Revolution and the growth of human activities. Environmental pollutants or toxins are defined as harmful substances in the surrounding environment that disrupt biological systems [14]. These pollutants—whether liquid, solid, or gaseous—can be transported through the air in large quantities, enabling their widespread dissemination without barriers [14]. Prolonged exposure to toxic substances, such as air pollutants, contributes to the development of numerous health conditions, including cancer and metabolic respiratory, neurological, and cardiovascular diseases [10–12, 14]. For example, endocrine-disrupting chemicals (EDCs), such as particulate matter (PM), heavy metals, and persistent organic pollutants (POPs), are environmental pollutants increasingly recognized as contributing factors in the development of metabolic disorders like obesity and diabetes [15]. This section highlights the influence of various environmental chemicals on the development and progression of DM.

### 2.1a Particulate Matter

PM is one of the most common global factors that threaten health and contribute to the development of many diseases [15, 16]. It consists of small air pollutants—microscopic solid particles or liquid droplets—composed of organic compounds, diesel exhaust, polycyclic aromatic hydrocarbons (PAHs), and reactive heavy metals. PM is classified into three categories based on particle diameter: PM10 (10 µm), PM2.5 (2.5 µm), PM1 (1 µm), and PM0.1 (0.1 µm). PM10 is less hazardous and is typically generated from sources, such as construction work and dust. In contrast, PM2.5, PM1, and PM0.1 are more dangerous because they can penetrate deep into tissues and the bloodstream. These finer particles are produced from both natural and man-made sources, such as vehicle exhaust and combustion activities. PM enters the body via inhalation and contributes to the development of several health conditions, including metabolic disorders like diabetes [17].

Zorena et al. [16] recently reported in a comprehensive review that exposure to PM2.5, PM10, and associated pollutants, such as nitrogen dioxide, sulfur dioxide, and heavy metals contributes to T1DM through oxidative stress, inflammation, and potential disruptions to the gut microbiome [16]. In addition, a longitudinal study involving more than 44,000 children and adolescents with T1DM was conducted to evaluate the association between PM10 and PM2.5 exposure and average hemoglobin A1c (HbA1c) levels—a marker of three-month cumulative blood glucose. The study demonstrated that PM exposure was strongly correlated with elevated HbA1c levels [18], possibly due to systemic inflammation, metabolic dysregulation, endothelial dysfunction, and dyslipidemia [18, 19]. For T2DM, a retrospective study spanning from 1990 to 2019 assessed the global burden trend of T2DM attributable to PM2.5 exposure and revealed a significant increase in low- and middle-income countries [20]. Furthermore, a systematic review and meta-analysis assessing the association between air pollutants and DM prevalence and incidence in developing countries found that long-term exposure to PM2.5 was linked to a 25% higher risk of developing T2DM, while no significant association was found with gestational diabetes throughout pregnancy [21]. These findings were further supported by Puett et al., who conducted a prospective epidemiological study investigating the relationship between PM exposure and T2DM incidence. While a weak association was found between PM exposure and T2DM overall, a slight increase in risk was observed in individuals living near roadways, particularly among women [22].

Several *in vivo* animal studies have investigated the effects of PM exposure and demonstrated an association with DM development. For example, Miranda et al. showed that daily exposure of Wistar rats to PM10 during pregnancy and the lactation period increased insulin levels and body weight, and altered pancreatic structure in male offspring. In contrast, female offspring appeared resilient to the adverse effects of maternal PM exposure, possibly due to the placenta’s enhanced adaptability against *in utero* environmental insults [23, 24]. In mice, exposure of male C57BL/6 mice to concentrated ambient PM2.5 for 10 weeks induced a non-alcoholic steatohepatitis like phenotype, characterized by hepatic lipid accumulation, inflammation, disrupted glucose regulation [25], adipose tissue inflammation, oxidative stress, and insulin resistance [26]. Collectively, these research findings suggest a link between PM exposure and an increased risk of DM.

### Heavy metals

Heavy metals are a group of high atomic weight elements that induce toxicity even at very low concentrations [15, 27]. Elements, such as arsenic (As), lead (Pb), mercury (Hg), and cadmium (Cd) are ranked among the most hazardous and toxic substances by the Agency for Toxic Substances and Disease Registry [28]. They commonly originate from natural, industrial, or agricultural sources, including drinking contaminated water or ingesting contaminated food [27, 29]. Heavy metals are not biodegradable and tend to accumulate in developing and industrialized cities, leading to disease development due to prolonged exposure and bioaccumulation in the human body [14].

As is one of the naturally occurring toxins found in the environment, present in either organic or inorganic forms, and has been associated with neurological disorders and respiratory complications [30–32]. In addition, a high load of as contributes to the progression and exacerbation of DM. This effect is mediated through alterations in pancreatic islet function and disruption of glucose uptake by inducing reactive oxygen species (ROS) and affecting Akt-related signaling pathways [30, 33]. An *in vivo* study on adult male NMRI mice exposed to different doses of As revealed a reduction in insulin secretion in pancreatic islets and increased oxidative stress in liver mitochondria [34]. Using the 3T3-L1 adipocyte cell line, it has been reported that treatment with toxic concentrations of As metabolites—such as trivalent arsenicals like arsenite, methylamine oxide, and iododimethylarsine—inhibits insulin-stimulated glucose uptake (ISGU) [35]. This diabetogenic effect of As metabolites is mediated through inhibition of the phosphorylation of 3-phosphoinositide-dependent kinase-1, protein kinase B, and AKT pathways, leading to impaired glucose tolerance [35]. In this context, it has been reported that activation of Akt plays a vital role in insulin signaling by promoting the translocation of glucose transporter type 4 (GLUT4) to the cellular membrane in response to insulin [35, 36].

Pb is another toxic heavy metal that is widely distributed in the environment due to both natural and anthropogenic sources, such as Pb-based paints, industrial emissions, and contaminated water [37]. It is highly persistent and non-biodegradable, leading to long-term contamination [38]. According to the ATSDR, Pb can cause severe health issues, including neurological, cardiovascular, hematological, and immunological disorders [38]. Blood Pb levels of approximately 5 µg/dL have been linked to neurological and developmental impairments, as well as cognitive deficits, especially in children [38, 39]. Importantly, exposure to Pb has also been strongly associated with an elevated risk of DM [29, 37]. A repeated-measures longitudinal study involving 5505 Chinese participants over a five-year follow-up period showed a significant association between blood Pb levels and increased fasting plasma glucose levels, along with reduced homeostatic model assessment of β-cell function (HOMA-B), an indicator of B-cell function [29, 40]. This association was predominantly observed in women, but not in men, suggesting a gender-dependent response [40]. Another human study, involving 110 industrial workers in the United Arab Emirates exposed to Pb in their workplace, demonstrated a positive association between blood Pb levels and fasting blood glucose and lipid levels, thereby increasing the risk of DM and heart disease [41]. In addition, an *in vivo* animal study on Wistar rats treated with various doses of Pb for 32 days showed marginally elevated blood glucose levels, fasting insulin levels, and HOMA-IR, a tool to measure insulin resistance [42]. Furthermore, pancreatic islet cells isolated from these Pb-treated rats exhibited reduced cell viability and impaired glucose-stimulated insulin secretion (GSIS), which was associated with increased ROS levels and elevated glycogen synthase kinase-3 beta (GSK-3β), contributing to insulin resistance [42].

### Organic pollutants

Organic pollutants are human-made, long-lasting, and toxic lipophilic substances that pose significant environmental and health concerns [43, 44]. These compounds are classified as either POPs or non-POPs (NPOPs), based on their ability to persist in the environment. While POPs are known for their long-term bioaccumulation, even NPOPs are toxic and can disrupt physiological pathways despite their relatively transient presence in biological systems [11, 45]. POPs include PAHs, halogenated aromatic hydrocarbons (HAHs), organochlorine pesticides, polychlorinated biphenyls (PCBs), and chemical by-products such as dioxins [45–47]. PAHs, such as benzo[a]pyrene B[a]P, and HAHs, such as 2,3,7,8-tetrachlorodibenzo-p-dioxin (TCDD) [11, 13, 28, 39], have prolonged half-lives and are commonly formed through cigarette smoking, the consumption of grilled and smoked foods, and the burning of fossil fuels [48].

The association between prolonged human exposure to POPs and an elevated risk of T2DM has been recently explored [44]. Ruzzin and his group reported that Sprague-Dawley rats fed for 28 days with a high-fat diet (HFD) containing POPs developed impaired insulin action, reduced ISGU in skeletal muscle and adipose tissue, and suppressed insulin-mediated glucose production in the liver compared to control rats [49]. These observations were confirmed *in vitro*, where treatment of differentiated 3T3-L1 adipocytes with a POP mixture for 48 h reduced ISGU [49]. A cross-sectional prospective study involving 2016 adults demonstrated a strong correlation between serum concentrations of POPs and T2DM risk, with organochlorines and PCB-153 showing the strongest association [50]. Similarly, a nested case-control study of 300 American Indians reported a positive, though not statistically significant, association between serum concentrations of POPs—particularly PCB-151—and the incidence and risk of diabetes [46]. An interesting study conducted on Chinese women investigated the impact of exposure to PAHs, kitchen ventilation, and exhaled nitric oxide (NO), revealing that prolonged inhalation of PAHs and NO fractions emitted during cooking was associated with an increased prevalence of T2DM among women who cook [51]. This highlights the role of occupational and environmental exposure in diabetes development [51]. This outcome may be mediated by the triggering of systemic inflammatory responses and oxidative stress, which disrupt insulin signaling pathways and glucose metabolism [28, 51]. Further supporting evidence comes from the study by Alshaarawy et al. [52], who analyzed 2769 participants in the National Health and Nutrition Examination Survey (NHANES) from 2001 to 2006 to examine the relationship between urinary monohydroxy-PAH (OH-PAH) metabolites and DM. The study found a positive association between specific PAH metabolites (e.g., 1-hydroxynaphthalene, 2-hydroxynaphthalene, and 2-hydroxyphenanthrene) and both HbA1c levels and DM prevalence [52]. Consistent with these findings, a study using Korean National Environmental Health Survey (KoNEHS) data from 2015 to 2017 demonstrated associations between PAHs (e.g., 2-hydroxyfluorene) and diabetes [53]. The study revealed that urinary concentrations of PAHs, along with the benzene metabolite trans, trans-muconic acid (t,t-MA), were positively associated with increased risk of DM and obesity [53]. While these findings collectively highlight the potential role of PAHs in the development of diabetes—and suggest that urinary PAH metabolites could serve as reliable biomarkers for DM—they also emphasize the need for further research to understand the causal relationship between PAHs and DM [51–53].

## Aryl hydrocarbon receptor (AHR) and DM

### AhR regulation

Several studies reinforce the hypothesis that exposure to environmental contaminants contributes to the development of DM. However, the underlying mechanisms and pathways by which these toxins impair glucose metabolism and induce insulin resistance remain unclear. In this context, previous studies have demonstrated that the toxic effects of environmental pollutants—such as HAHs and PAHs—are mediated through the activation of a transcription factor, the AhR [9, 13, 54, 55]. AhR is a xenobiotic-activated cytosolic transcription factor expressed in various body tissues, with the highest levels found in the liver, lungs, bone marrow, urinary tract, and gall bladder [13]. In pancreatic cells, AhR is expressed at varying levels. Studies have shown that AhR exhibits low expression in normal pancreatic tissue, with predominant localization in acinar and ductal cells. However, its expression is moderately elevated in conditions such as chronic pancreatitis and significantly upregulated in pancreatic cancers, highlighting its potential role in pancreatic cell responses and disease pathophysiology [56–58]. AhR is an active member of the basic-helix-loop-helix (bHLH)/Per-ARNT-Sim (PAS) family, which has regulatory roles in differentiation, proliferation, and tumor growth [11, 54, 59]. In addition, it induces the transcription and expression of certain cytochrome P450 family 1 (CYP1) enzymes, including CYP1A1, CYP1A2, and CYP1B1, which are responsible for metabolizing xenobiotics into highly reactive intermediates [54, 60, 61]. Studies have reported the mechanistic role of AhR in various medical conditions, such as cancer, cardiovascular diseases, autoimmune disorders, and metabolic disorders [10, 54, 55, 61–64]. AhR is a target for multiple ligands—some endogenous, such as tryptophan and indole-derived amino acid metabolites, and others plant-derived, such as resveratrol [9, 11, 65]. In addition to these, exogenous triggers—including PAH molecules like B[a]P and dioxins such as TCDD—exist in the air and food as complex mixtures. These compounds are highly stable and tend to accumulate in adipose tissues [9, 66].

**Figure 1.**
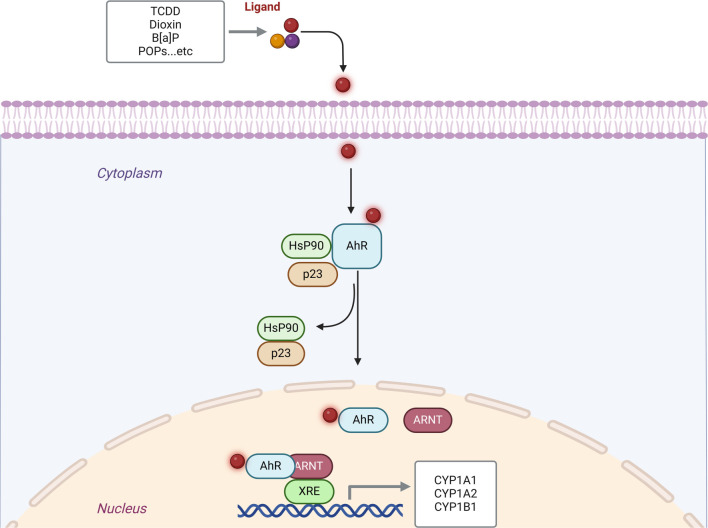
**AhR/CYP1 signaling pathway: Activation of the AhR is initiated through binding to its ligands, such as TCDD, B[a]P, or PoPs.** Ligand-induced conformational shifts of AhR lead to its nuclear translocation, forming a complex with ARNT, and subsequent binding to xenobiotic response elements in regulatory regions of the genome. This regulates the expression of target genes, such as CYP1A1, CYP1A2, and CYP1B1. CYP1: Cytochrome P450 family 1; AhR: Aryl hydrocarbon receptor; Hsp90: Heat shock protein 90; ARNT: AhR nuclear translocator; XRE: Xenobiotic response elements.

A series of molecular events is activated following xenobiotic binding to the AhR receptor in the cell cytoplasm (Figure 1). Initially, the receptor dissociates from its inhibitory proteins—heat shock protein 90 (HSP90) and p23—allowing its translocation into the nucleus. Once in the nucleus, the activated AhR receptor heterodimerizes with the transcription factor AhR nuclear translocator (ARNT). The AhR–ARNT complex then binds specifically to the xenobiotic response element (XRE) on the DNA of target genes, such as those of the CYP1 family, initiating their transcription [54, 59]. Since these environmental toxins depend on AhR to exert their toxic effects, AhR is likely to play a role in diabetes development, insulin resistance, and glucose homeostasis.

### Role of AhR in glucose metabolism and insulin resistance

The bioactivation of AhR and its regulated genes—CYP1A1, CYP1A2, and CYP1B1—plays a role in metabolic functions involving glucose homeostasis, insulin sensitivity, and lipid metabolism [67]. Many studies have demonstrated an association between the expression of AhR/CYP1 genes and diabetes-related dysfunctions. It has been reported that chronic exposure to low levels of dioxins is associated with insulin resistance and the development of T2DM [68]. Moreover, epidemiological studies have revealed that dioxin-mediated activation of AhR is linked to a higher risk of T2DM and obesity [60]. The Brazilian Longitudinal Study of Adult Health (ELSA-Brazil), conducted in 2061 participants, demonstrated a significant increase in the incidence of DM among those with higher AhR ligand bioactivity [67].

In animal studies, it has been shown that injecting wild-type (WT) and AhR-knockout (KO) C57BL/6J mice with TCDD—a potent AhR/CYP1 inducer—caused impaired GSIS, decreased plasma insulin levels, and inhibited insulin response in WT mice compared to the KO group [69, 70]. In contrast, AhR KO mice showed enhanced insulin sensitivity and improved glucose tolerance compared to WT mice [69, 70], indicating an AhR-dependent mechanism. In addition, Xu et al. [69] demonstrated that AhR-deficient mice were protected against HFD-induced obesity, insulin resistance, inflammation, and hepatic steatosis. Similarly, Liu et al. [71] reported that deficiency of CYP1B1 in C57BL/6J mice prevented HFD-induced obesity and glucose intolerance in adult mice compared to WT mice, suggesting an important role of CYP1B1 in energy metabolism and insulin sensitivity. An additional animal study in zebrafish showed that 24–48 hpf (hours post-fertilization) zebrafish embryos exposed to PCB-126 at concentrations of 2–5 nM exhibited pancreatic islet dysmorphology, resulting in the manifestation of ectopic β-cells and islet fragmentation [72]. Collectively, these animal studies suggest a possible role of AhR/CYP1 activation in glucose intolerance, impaired insulin secretion, pancreatic islet dysfunction, and, consequently, the development of DM. One of the possible underlying mechanisms is the ability of AhR activation to inhibit effector T cell function while inducing regulatory T cells (Tregs), which may contribute to pancreatic β-cell dysfunction and the subsequent development of DM [13]. This hypothesis is supported by the fact that AhR and its target genes are expressed in both innate and adaptive immune cells, as well as anti-inflammatory cells, which are involved in immune responses and the regulation of inflammatory cytokine secretion—factors that may influence the autoimmune reaction seen in T1DM [73]. At the *in vitro* cell line level, it has been reported that exposure of human hepatoma HepG2 cells and multiple rodent pancreatic endocrine cell lines (MIN6, βTC-6, INS1, α-TC1, α-TC3) to the AhR activator TCDD leads to increased β-cell death, suppression of insulin secretion, and subsequently reduced plasma insulin levels [62].

## Crosstalk between AHR/CYP1 pathway and epigenetic modifications in insulin resistance and glucose hemostasis

While previous studies and reviews have highlighted several mechanisms that mediate the involvement of the AhR/CYP1 pathway in the pathogenesis of DM [13], to our knowledge, there is a scarcity of research and a lack of clear understanding regarding the crosstalk between the AhR/CYP1 pathway and epigenetic modifications in the context of insulin resistance and diabetes.

**Figure 2.**
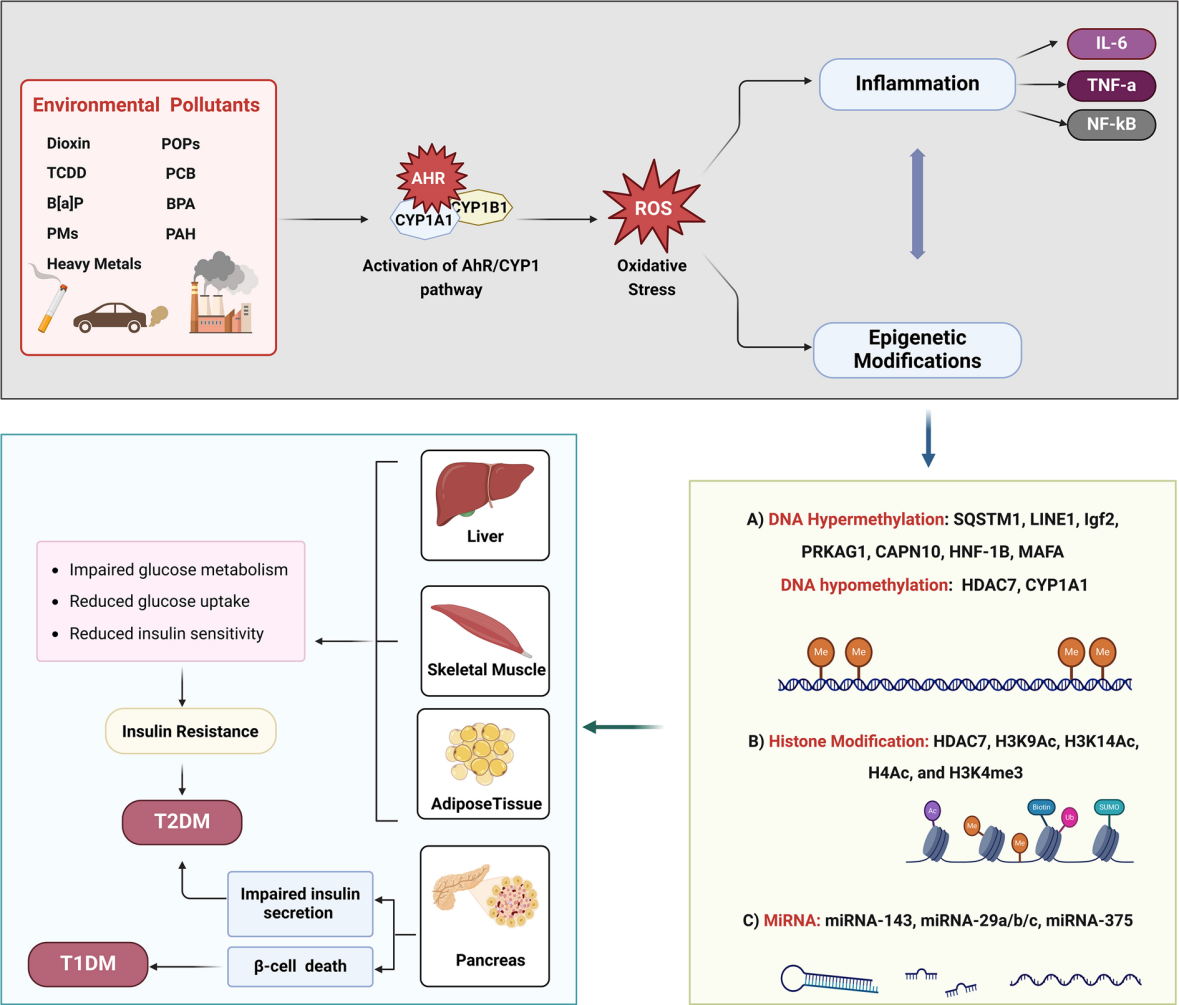
**Schematic diagram highlights how environmental pollutants trigger epigenetic modifications and activate the AhR/CYP1 pathway, contributing to the development of insulin resistance and increased diabetes risk.** Environmental pollutants, such as dioxins and heavy metals activate AhR/CYP1, leading to oxidative stress and systemic inflammation (e.g., IL-6, TNF-α, and NF-kB). This results in aberrant DNA methylation (e.g., *SQSTM1, LINE-1,* and *Igf2*), histone modifications (e.g., HDAC7 and H3K9Ac), and miRNA dysregulation (e.g., miRNA375 and miRNA-143). These epigenetic changes disrupt insulin signaling and glucose metabolism and contribute to DM. CYP1: Cytochrome P450 family 1; AhR: Aryl hydrocarbon receptor; SQSTM1: Sequestosome 1; LINE-1: Long interspersed nuclear element-1; Igf2: Insulin-like growth factor 2; miRNA: MicroRNA; DM: Diabetes mellitus; NF-kB: Nuclear factor kappa-B.

Epigenetics studies the complex interaction between environmental stimuli, genetics, and the onset of diseases [74]. Disease vulnerability depends on the interplay between individual genetic profiles and epigenetic modifications influenced by environmental factors [75]. It explores reversible, heritable alterations in gene expression that occur independently of changes in the original DNA sequence, leading to the misinterpretation of genes by the cell. The three primary recognized epigenetic mechanisms are DNA methylation, histone modifications, and non-coding RNA (ncRNA) [2, 6]. Environmental pollutants are well known to mediate epigenetic changes involved in the development of various diseases, including metabolic disorders and diabetes [74, 76]. These pollutants increase the production of ROS, which leads to DNA damage, altered methylation, and disruption of multiple inflammatory signaling pathways, including the nuclear factor kappa-B (NF-κB). This, in turn, results in metabolic dysregulation, including insulin resistance, glucose intolerance, and dyslipidemia [15, 77]. This section explores the possible links between epigenetic alterations and AhR/CYP1 activation by environmental toxins in contributing to the risk of insulin resistance and diabetes, as summarized in Table 1 and Figure 2.

### DNA methylation

DNA methylation is one of the most important epigenetic mechanisms involved in many disorders, including cardiovascular disease, obesity, cancer, and diabetes [9, 66, 74]. During the methylation process, a methyl group is transferred by DNA methyltransferase enzymes (DNMTs) and covalently binds to the cytosine of CpG dinucleotides within CpG islands, which are typically found in 5’ regulatory regions, such as promoters, intragenic areas, and enhancers [78]. Hypermethylation is generally associated with gene silencing, whereas hypomethylation leads to increased gene expression [7, 78–80].

DNA methylation has been reported to be induced by various environmental pollutants, including bisphenol A (BPA), PAHs, PM, and heavy metals [9, 74]. A genome-wide study in 400 adult individuals revealed a significant association between blood and urine As levels and gene-specific DNA methylation [81, 82]. Higher As exposure was linked to reduced methylation levels at the Sequestosome 1 *(SQSTM1*) gene, which encodes the sequestosome-1 protein that binds ubiquitin and regulates activation of the NF-κB pathway [81]. Additionally, *SQSTM1* has been implicated in several diseases, including insulin resistance. These findings suggest that toxicity could be mediated by epigenetic modifications, particularly DNA methylation [81].

**Table 1:** Impact of the AhR-activating environmental pollutants on DM

**Study models**	**Types**	**Toxic substance**	**Epigenetic mechanisms**	**Affected gene**	**Impact in diabetes**	**Ref.**
*Human*		As	DNA hypomethylation	SQSTM1	***↑*** insulin resistance	[81]
		PM	DNA methylation /demethylation		***↑*** ROS production, ***↑*** glucose intolerance ***↑*** dyslipidemia	[83]
		PM, POPs, and PBDEs	DNA methylation	LINE-1	***↑*** incidence of T2DM	[82]
*In vivo*	F1 hybrid progeny from C57BL/6(B6), and CAST/(C7)	BPA (prenatal exposure)	DNA methylation	Igf2	***↓*** Igf2 imprinting ***↓***β-cell development ***↓*** glucose tolerance	[74, 87]
	C57BL/6 mice (Liver)	TCDD	DNA demethylation	Cyp1a1	***↑***metabolic dysregulation, ***↑***glucose intolerance	[15, 75, 84, 90]
*In vitro*	hESCs cell line	TCDD	DNA hypermethylation	PRKAG1, CAPN10, HNF-1B MAFA	***↓*** pancreatic lineage differentiation ***↑*** risk of T2DM	[88]
		TCDD	DNA hypomethylation	HDAC7	***↑***β-cell dysfunction ***↓*** insulin secretion	[88]
	HepG2 cell line	TCDD	Histon modification (H3K9Ac, H3K14Ac, H4Ac, and H3K4me3)	CYP1A1 and CYP1B1	***↑*** incidence of DM	[92, 105]
	pHBEC cell lines	PM and DEPs	miRNA-375	AhR	***↑*** incidence of DM	[116]

As: Arsenic; PM: Particulate matter; miRNA: MicroRNA; CYP1: Cytochrome P450 family 1; AhR: Aryl hydrocarbon receptor; SQSTM1: Sequestosome 1; LINE-1: Long interspersed nuclear element-1; Igf2: Insulin-like growth factor 2; DM: Diabetes mellitus; TCDD: 2,3,7,8-tetrachlorodibenzo-p-dioxin; T2DM: Type 2 DM; pHBEC: Primary human bronchial epithelial cell; H3K4me3: Trimethylation of lysine 4 on histone H3; HNF-1B: Hepatocyte nuclear factor 1 homeobox B; PRKAG1: Protein kinase AMP-activated non-catalytic gamma 1; CAPN10: Calpain-10; hESC: Human embryonic stem cell; ROS: Reactive oxygen species.

A recent paper has linked exposure to environmental pollutants, particularly PM10, with DNA methylation of genes related to cardiovascular diseases, respiratory diseases, immune responses, and oxidative stress [83]. In the context of oxidative stress, it has been reported that increased production of ROS can lead to epigenetic alterations in nuclear methylation/demethylation activity, either by modifying the sites of methylated CpG or by altering enzymatic expression [15, 83]. A human study reported that exposure to air pollutants during the first trimester was associated with DNA hypomethylation of the long interspersed nuclear element-1 (*LINE-1*) gene in newborns, whereas hypermethylation was observed during the later stages of pregnancy [82, 84]. The epigenetic alteration of the *LINE-1* gene in response to environmental pollutant exposure influences glucose metabolism and is associated with a higher risk of DM during the developmental stage [84–86]. An animal study demonstrated that exposure to EDCs, such as BPA, during prenatal development disrupts the genetic imprinting of genes like insulin-like growth factor 2 (*Igf2*), a critical regulator of early pancreatic β-cell development [74, 87]. Prenatal BPA exposure increases DNA methylation of the Igf2 differentially methylated region 1 (DMR1), which is normally an unmethylated regulatory region. This prevents the binding of the GC-binding factor 2 (GCF2) repressor protein and permits activation of *Igf2* expression from both maternal and paternal alleles. The loss of imprinting leads to abnormal *Igf2* expression, which may hinder β-cell maturation and lead to glucose intolerance later in life [74, 87]. Kubi et al. investigated early embryonic exposure to low-dose TCDD and alterations in the DNA methylome on early pancreatic lineage development. Using human embryonic stem cells (hESCs) as a model to mimic early pancreatic development, they reported that treatment with TCDD (10 and 100 pM) for two weeks disrupted DNA methylation profiles, causing hypermethylation of key genes involved in pancreatic development and function, such as protein kinase AMP-activated non-catalytic gamma 1 (*PRKAG1*), Calpain-10 (*CAPN10*), and hepatocyte nuclear factor 1 homeobox B (*HNF-1B*) [88]. These epigenetic alterations negatively affected the differentiation of hESCs into pancreatic progenitor cells and reduced the expression of critical developmental markers, such as Sex-determining region Y-box 17 (*SOX17*), forkhead box A2 (*FOXA2*), and pancreatic and duodenal homeobox 1 (*PDX1*) [88]. For example, the hypermethylation of *PRKAG1* persisted throughout differentiation stages, indicating that initial TCDD exposure can induce permanent epigenetic changes and increase the risk of T2DM later in life [88]. Linking these observations to the AhR/CYP1 pathway, it has been reported that 24-h exposure to TCDD induced demethylation of two CpG sites at the Cyp1a1 proximal promoter in the liver of C57BL/6J mice [75, 89]. This, in turn, results in metabolic dysregulation, including glucose intolerance and dyslipidemia [15, 83]. These findings suggest a novel crosstalk between AhR and DNA methylation in the regulation of glucose metabolism and insulin secretion. Further experimental studies are encouraged to explore this interaction and the underlying molecular mechanisms.

### Histon modification

Histones are a group of globular proteins—including H1, H2A, H2B, H3, and H4—that surround DNA to form chromatin. Conformational changes in the structure of post-translational histones may occur due to enzymatic modifications of lysine and arginine residues at the amino terminus [78]. These alterations—such as methylation, acetylation, ubiquitination, lactylation, or phosphorylation—regulate the silencing or expression of specific genes, depending on the type of modification [7, 78, 90]. For example, trimethylation of lysine 4 on histone H3 (H3K4me3) is associated with gene expression, whereas dimethylation of lysine 9 (H3K9me2) leads to gene silencing [91]. H3K4me3, in particular, is essential for maintaining the functions of pancreatic β-cells, and its remodeling is associated with gene expression changes implicated in the pathogenesis of diabetes [92]. It has been reported that overexpression of the *Fxyd3* gene, an FXYD domain-containing ion transport regulator 3 gene, reduces glucose-induced insulin secretion from β-cells in diabetic mice [93]. In this context, a chromatin immunoprecipitation (ChIP) assay revealed that upregulation of H3K4me3 at the transcriptional start site of the Fxyd3 gene in the islets of *Glp1r-/-; Gipr-/-* double KO (dKO) mice was associated with reduced expression of the *Fxyd3* gene [93]. Another ChIP study involving H3K4me3, H3K27me3, H3K9me3, H3K9Ac, and H4K16Ac showed elevated levels of H3K9Ac in T1DM-susceptible genes—such as HLA-DRB1 (HLA class II histocompatibility antigen, DRB1 β chain) and HLA-DQB1—in T1DM patients compared to a healthy cohort [94]. Moreover, acetylation of the *FOXO1* gene, which regulates PDX1, plays a role in pancreatic β-cell development and glucose homeostasis. Furthermore, it has been reported that deacetylation of histone H3 lysine 9 (H3K9) via histone deacetylase 6 (HDAC6) leads to suppression of insulin receptor substrate 2 (IRS2) protein, which in turn contributes to the development of insulin resistance [8, 95].

Histone lysine lactylation (Kla) is a novel post-translational histone modification that was first recognized in 2019 [96–99]. It involves the addition of a lactyl group to the lysine residue of the histone protein, primarily on H3 and H4, such as H3K18la, H3K14la, and H4K12la [96, 100]. This modification is derived from lactate, a byproduct of glycolysis, which has been implicated in various biological processes [96, 100]. Similar to other histone modifications, histone Kla activates gene expression and is involved in the progression of various diseases, including cancer [101, 102], cardiovascular disorders [96, 99, 103], and insulin resistance [104]. A recent study involving 15 lean and 14 obese adults who underwent oral glucose tolerance tests and muscle biopsies showed that higher levels of lactylation occur in the skeletal muscle of obese individuals, particularly females. These findings were further supported by *in vitro* experiments using human skeletal muscle cells (HSkMCs), which demonstrated that lactate exposure led to a dose-dependent increase in IRS-1 serine phosphorylation—a marker of insulin resistance [104].

In correlation with the AhR/CYP1 pathway, Kubi et al. [88] demonstrated an association between the upregulation of HDAC7 and β-cell dysfunction and impaired insulin secretion in hESCs treated with a low dose of the AhR inducer TCDD, suggesting that inhibition of HDAC7 could be a promising targeted therapy for treating T2DM. In addition, a ChIP assay conducted in HepG2 and human breast cancer MCF-7 cells treated with 100 nM TCDD showed the induction of various histone modifications, including H3K9Ac, H3K14Ac, H4Ac, and H3K4me3 at the CYP1A1 and CYP1B1 promoters in MCF-7 cells, and at the CYP1A1 promoter region in HepG2 cells only [92, 105]. Given that histone modifications involving H3K4me3 and H3K9Ac are associated with changes in gene expression relevant to diabetes—and that the AhR/CYP1 pathway also induces modifications of specific histones, such as H3K9Ac and H3K4me3—it is plausible that histone-mediated epigenetic regulation may serve as a mechanistic link between AhR/CYP1 activity and β-cell dysfunction in diabetes. However, further studies are needed to explore the pathways and underlying mechanisms in greater detail.

### MicroRNAs (miRNA)

Non-coding miRNA consists of 22 nucleotides of single-stranded RNA [106]. miRNAs are among the most common classes of molecules involved in gene expression regulation and control various biological activities. In addition, they regulate the expression of many protein-coding genes by targeting mRNAs for cleavage or by causing translational repression. Dysregulation of miRNAs has been observed in numerous diseases, including Alzheimer’s disease, diabetes, and cancer. DNA methylation, histone modifications, and RNA modifications can all regulate the activity of miRNAs [80, 107].

A genome-wide study of miRNA expression in patients with T1DM revealed a significant increase in miR-510 and a reduction in miR-342 and miR-191 levels compared with the non-diabetic group [4]. In addition, elevated miR-326 levels were found in the peripheral blood lymphocytes of T1DM patients, which were directly associated with disease severity [108]. A case-control study involving 326 patients with T2DM and 342 healthy controls explored the association between two genetic variants of miR-143 (rs4705342 and rs353292) and the risk of T2DM in the Chinese population. In this study, higher serum expression levels of miR-143 were observed in subjects with the CC genotype of rs4705342, which was associated with elevated levels of low-density lipoprotein cholesterol (LDL-C), fasting blood glucose (FBG), HbA1c, and an increased risk of T2DM [109]. These results support a role for the rs4705342 CC genotype of miR-143 in the pathogenesis of T2DM through increased miR-143 expression, suggesting its potential as a biomarker and therapeutic target for T2DM [109].

The miR-29 family, which includes miR-29a, miR-29b1, miR-29b2, and miR-29c, plays a negative role in glucose tolerance and insulin resistance [110]. While the miR-29 family is expressed in many organs—such as the liver, adipose tissue, and skeletal muscles—it is also among the most abundant miRNAs expressed in the pancreatic cells of NOD mice. Overexpression of miR-29 has been shown to downregulate GSIS in primary islet cells of mice [111]. In Goto-Kakizaki diabetic rats, elevated levels of miR-29 family members have been associated with insulin resistance in skeletal muscle, liver, and adipose tissue [112]. Additionally, transfection of 3T3-L1 adipocytes with adenovirus-mediated overexpression of miR-29a/b/c resulted in significant repression of ISGU through deactivation of the Akt signaling pathway [112].

On the other hand, miR-375 has been shown to be the most abundantly expressed miRNA in pancreatic β-cells, where it is essential for the regulation of insulin secretion [113]. Data from an experimental study using miR-375 KO and miR-375/obese KO mice revealed hyperglycemia due to an increase in pancreatic α-cell mass, impaired glucose tolerance, and reduced insulin secretion—hallmarks of insulin resistance in the miR-375 KO group [114]. A study by Kumar et al. [115] showed that ginger-derived nanoparticles upregulated miR-375 and markedly improved glucose tolerance and insulin sensitivity. This miRNA inhibited the overexpression of AhR and decreased the production of the AhR ligand indole from gut bacteria. Additionally, miR-375 targeted genes involved in hepatic insulin signaling, resulting in improved systemic insulin sensitivity. These findings highlight the importance of miR-375 in gut and liver homeostasis and suggest that it may serve as a novel therapeutic target for the treatment of metabolic disorders [115].

In a separate experimental study, researchers explored the relationship between miR-375 and AhR expression in airway epithelial cells exposed to air pollutants, including diesel exhaust particles (DEPs) and PM [116]. The study found that treatment with DEP or PM in primary human bronchial epithelial cells (pHBECs) resulted in significant overexpression of miR-375 and thymic stromal lymphopoietin (TSLP), suggesting that miR-375 may negatively regulate AhR. Additional validation using a miR-375 mimic showed a modest but statistically significant decrease in AhR mRNA levels in pHBEC. Notably, DEP exposure inhibited AhR expression in pHBEC, an effect that was reversed through anti-miR-375 transfection, identifying miR-375 as a regulator of AhR. These results suggest that air pollutants induce upregulation of miR-375 and TSLP via the AhR pathway in pHBEC [116].

To the best of our knowledge, the association between environmental pollutant exposure and miRNA expression—specifically in the context of diabetes—remains an unexplored area. Collectively, studies suggest that pollutants may chronically upregulate miR-375, potentially disrupting its regulatory role in pancreatic cells and contributing to diabetes pathogenesis. Additionally, epigenetic variations appear to correlate with AhR/CYP1 pathway-related gene expression, highlighting a complex dual relationship. However, the precise mechanisms linking the AhR/CYP1 pathways, epigenetic modifications, and glucose intolerance remain unclear. Therefore, further detailed investigations are needed to unravel these interactions and their role in the development of diabetes.

## Conclusion

This review underscores the critical role of environmental toxins in the pathogenesis of DM through epigenetic modifications and AhR activation. It supports evidence linking environmental exposures—such as heavy metals, air pollutants, and POPs—with disruptions in glucose homeostasis and insulin resistance. The AhR/CYP1 pathway emerges as a central player, mediating the diabetogenic effects of these pollutants and influencing DNA methylation, histone modifications, and miRNA expression. Although significant advancements have been made in understanding these pathways, the precise mechanisms underlying their interplay remain incompletely understood. Future research focusing on this critical intersection could enhance our understanding of diabetes etiology and drive the development of innovative therapeutic strategies to address the effects of environmental pollutants on metabolic health.

## Data Availability

The data is available upon request.
